# Severe Combined Immunodeficiency with De Novo Duchenne Muscular Dystrophy Mutation

**DOI:** 10.1097/PG9.0000000000000135

**Published:** 2021-11-29

**Authors:** Kevin P. Shah, Vignesh Ramachandran, Sarah K. Nicholas, Imelda C. Hanson, Timothy E. Lotze, Caridad A. Martinez, Douglas S. Fishman

**Affiliations:** From the * Baylor College of Medicine, Houston, TX; † Section of Allergy and Immunology, Texas Children’s Hospital, Houston, TX; ‡ Section of Neurology, Texas Children’s Hospital, Houston, TX; § Section of Hematology-Oncology, Texas Children’s Hospital, Houston, TX; ∥ Section of Gastroenterology, Hepatology and Nutrition, Texas Children’s Hospital, Houston, TX.

**Keywords:** elevated transaminases, primary immunodeficiency, myopathy, bone marrow transplant, graft vs host disease

## Abstract

Both severe combined immunodeficiency (SCID) syndrome and Duchenne muscular dystrophy (DMD) are rare conditions. Patients with X-linked SCID have pathogenic variants of the *IL2RG* gene, resulting in defective cellular and humoral immunity. DMD is also an X-linked condition caused by a *dystrophin* gene mutation, causing progressive proximal muscle weakness. We present a patient diagnosed with SCID at birth who underwent matched unrelated donor bone marrow transplant (BMT). Several months after, he was noted to have persistently elevated aminotransferases. Despite a lack of clinical signs of graft versus host disease (GvHD), a liver biopsy revealed mild GvHD. Creatine kinase (CK) levels of >19,000 U/L prompted evaluation for muscular dystrophies. Given BMT, genetic analysis was not an option. Muscle biopsy confirmed DMD. This case highlights the complexity of diagnosing and managing uncommon genetic conditions through a multidisciplinary team-based approach. This case is only the second reported case of SCID and DMD together.

## INTRODUCTION

Severe combined immunodeficiency (SCID) is a rare genetic condition inherited in an autosomal recessive or X-linked inheritance pattern. It is characterized by absent T cells, resulting in defective humoral and cellular immunity. Patients with X-linked SCID have pathogenic variants of the *IL2RG* gene, which encodes cytokines that are essential in the development and maturation of functional lymphocytes (1). Historically, patients with X-linked SCID presented in infancy with chronic diarrhea, severe recurrent infections, and failure to thrive. In some well-appearing newborns, early diagnosis was delayed due to circulating protective maternally derived antibodies (2). However, many cases of SCID are now detected before onset of infection through newborn screening, now mandatory in all 50 states (3). Once SCID is suspected, the diagnosis is confirmed with lymphocyte eneumeration and proliferation testing as well as genetic confirmation. Earlier diagnoses and treatment are associated with improved outcomes and survival in numerous studies (4). The current standard of care for SCID is hematopoietic stem cell transplant (3).

Duchenne muscular dystrophy (DMD) is a severe, progressive disorder affecting 1 in 3,600 male newborns. DMD causes proximal muscle weakness, delayed motor milestones, and eventual loss of ambulation by adolescence. An X-linked disease, DMD is characterized by genetic mutations (usually deletions) affecting the *dystrophin* gene. This results in muscle necrosis and degeneration. There are many identified mutations of DMD with variable phenotype expressivity, which can delay diagnosis especially since patients have normal development early in life. Without a known family history, suspicion of DMD arises with abnormal motor function in a male child who clinically may present with difficulty walking, frequent falling, labored respiration, or Gower’s sign. Laboratory markers such as creatine kinase are often elevated. Confirmation of diagnosis requires testing for mutations in DMD from a blood sample. Muscle biopsy is less often necessary with current genetic methods but can be performed to confirm the complete absence of *dystrophin* protein expression. The current standard of care for patients with DMD is limited to glucocorticoids which delay loss of ambulation—without a cure (5).

Herein, we present a complex case of SCID and DMD, requiring multidisciplinary care coordination and management.

## CASE REPORT

**FIGURE 1.**
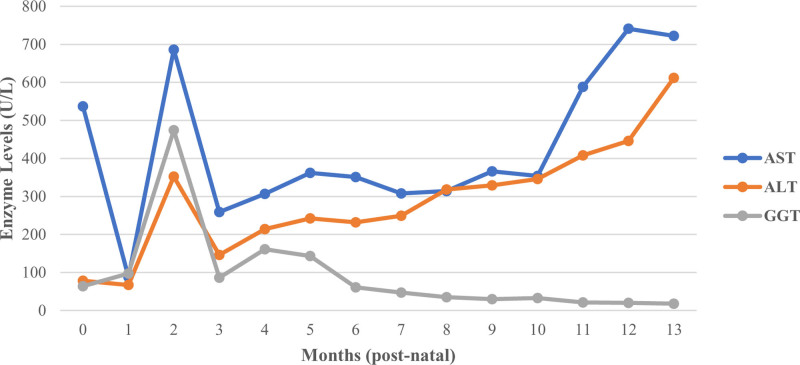
This shows the trend of liver indices that led to the initial biopsy with suspicion of GvHD and eventual diagnosis of DMD at 11 months. DMD indicates Duchenne muscular dystrophy; GvHD, graft versus host disease.

An 8-month-old male with history of matched unrelated donor (MUD) bone marrow transplant (BMT) 6 weeks after birth for SCID was referred to gastroenterology with persistent elevation of aminotransferases (Fig. 1). History of present illness and review of systems from family were unrevealing. Due to a family history of SCID (two older brothers), the patient underwent newborn testing for SCID with targeted gene sequencing probed for certain *ILR2RG* regions believed to harbor the X-linked familial variant. This revealed hemizygous, recessive X-linked SCID with c.184T>C (p.cys62Arg) missense mutation in the *IL2RG* gene, the same novel mutation in *IL2RG* as his two siblings.

Physical exam at presentation was unremarkable. The patient underwent comprehensive evaluation of elevated transaminases, which was negative for alpha-1 antritrypsin deficiency, autoimmune hepatitis, and other common etiologies. Given his history of BMT, there was concern that elevated transminases may be due to graft versus host disease (GvHD). Pathology revealed patchy minimal lobular hepatitis, suggestive of mild acute GvHD. Post-BMT immunosuppression regimen was escalated accordingly.

However, the patient continued to have persistent elevation of ALT and AST at follow-up with further testing revealing creatine kinase levels of greater than 19,000 U/L (normal 60–305 U/L). This prompted immediate referral to neurology to evaluate for muscular dystrophy. At that time, physical exam was notable for intact cranial nerves, decreased motor tone, mild head lag, calves firm to palpation with mild hypertrophy, and ability to bring himself to a seated/standing position with hands held. Western blot of a muscle biopsy sample demonstrated normal molecular size of dystrophin, but in a quantity less than 5% of normal, a finding consistent with DMD. There was no evidence of GvHD manifestation in the muscle tissue sample. Given his history of bone marrow transplant, peripheral lymphocytes could not be utilized for genetic analysis. Therefore, the muscle biopsy was utilized for *DMD* mutation analysis and identified a IVS25-1G>A splice site mutation at exon 25/26 boundary via gene sequencing.

There was no known family history of neuromuscular disorders. The family received genetic counseling for both SCID and DMD to evaluate for identification of other at-risk family members. The patient was initiated on steroids and was referred for therapy services including physical, occupational, and speech therapy.

## DISCUSSION

We present a case of SCID secondary to novel mutation in *IL2RG* and DMD caused by *de novo* mutation. Combined SCID and DMD is extremely rare with an incidence of 1 out of 7 million people and our literature review revealed only one other such case (6). This case also highlights the consideration of muscular dystrophies and myopathies in the setting of elevated aminotransferences given the expression of these enzymes in muscle cells.

Early diagnosis of SCID and DMD is critical to provide necessary interventions and prevent adverse outcomes. Chan et al estimated a gap of 3.5 months between onset of SCID symptoms and formal diagnosis of SCID in the absence of a family history. Moreover, newborns tested for SCID at birth had 85% survival compared to 58% survival of those not tested at birth (7). Newborn screening for SCID has become mandatory in all 50 US states over the last decade (3). However, screening for DMD lags behind, varying significantly from state to state (8). Gatheridge et al evaluated CK levels in over 1.8 million newborns and found 344 positive for DMD and 80 positive for non-DMD muscular disorders (9). In this particular case, screening of CK levels may have allowed for quicker diagnosis, prognostic, and genetic counseling, as well as initiation of age-appropriate guideline-based treatment for DMD.

Uncommon genetic conditions in pediatric populations can be difficult to diagnose and may require complex medical care (10). The use of common laboratory tests have the potential to raise clinical suspicion of even rare diagnoses. When elevation of AST and ALT are noted, it is important to check GGT and CK levels. In this particular case, persistently elevated aminotransferases and CK levels raised suspicion for an etiology beyond mild GvHD and led to the diagnosis of DMD. Ultimately, the management of complex medical cases with systemic manifestations, as seen in this patient with SCID and DMD, necessitates care coordination with a multidisciplinary team-based approach.
